# Effects of Apical Dendrite Integrity on Action Potential Properties in Layer 6 Corticothalamic Pyramidal Neurons: Evidence for Hemispheric Differences

**DOI:** 10.3390/biology15080608

**Published:** 2026-04-12

**Authors:** Ahmed A. Aldohbeyb

**Affiliations:** Department of Biomedical Technology, College of Applied Medical Sciences, King Saud University, Riyadh 12372, Saudi Arabia; aaldohbeyb@ksu.edu.sa

**Keywords:** pyramidal neurons, corticothalamic, layer 6, action potential, hemispheric asymmetries, rapidity, threshold, apical dendrite

## Abstract

Hemispheric asymmetries in the cortical structure and function are well documented, but whether this asymmetry is reflected in the electrophysiological properties of the same neuron type remains unclear, particularly given the many factors that influence action potential (AP) generation. Among these factors, dendritic tree size plays a crucial role in AP generation and in forward and backward propagation, yet it is not well understood whether dendritic size affects spike generation similarly in the two hemispheres. Here, electrophysiological recordings of layer 6a corticothalamic (CT) pyramidal neurons from a mouse’s left and right primary visual cortex were analyzed to examine how apical dendrite integrity influences somatic AP properties. Neurons were divided into four groups based on hemisphere and apical dendrite status (intact vs. truncated), and the AP shape parameters were quantified in response to current stimulation. Intact CT neurons showed similar AP rapidity and width across hemispheres but differed in AP threshold and amplitude. In contrast, a comparison of the intact and truncated neurons within each hemisphere revealed significant and opposing effects of apical dendrite truncation on all AP parameters. These results suggest that the coupling between dendritic morphology and somatic AP generation differs between hemispheres, implying distinct rules for integrating dendritic load in the left and right CT neurons.

## Simple Summary

The brain is divided into left and right hemispheres that often differ in structure and function, but it is less clear whether the same neuronal cell type follows different rules on each side. In this study, a specific population of deep-layer pyramidal neurons in a mouse’s primary visual cortex, which project to the thalamus, was examined with a focus on how the integrity of their apical dendrites shapes action potential properties. By comparing neurons from the left and right hemispheres with either intact or truncated apical dendrites, I found that the baseline spike shape was largely similar between hemispheres, but that the apical dendrite truncation produced opposite effects on the action potential of the onset dynamics on the two sides. These findings indicate that the relationship between the dendritic structure and spike generation may not be identical in the left and right visual cortex, and point to subtle hemispheric differences that merit further investigation.

## 1. Introduction

The primary visual cortex (V1) is the first region of the visual cortex that receives and processes information [1]. As for the rest of the neocortex, V1 is divided into a six-layered laminar organization. In layer 6 (L6), neurons in V1 form a closed corticothalamic loop by receiving direct input from the lateral geniculate nucleus (LGN), sending feedback projections back to the LGN, and giving recurrent axon collaterals to layer 4 within V1 [2,3,4]. As a result, neurons in L6 are highly diverse and strategically positioned to regulate neuronal activity [5].

Pyramidal neurons in L6 comprise diverse subtypes that are commonly grouped into three major classes: corticothalamic (CT), corticocortical (CC), and corticoclaustral pyramidal neurons [4]. A key distinction between L6 pyramidal neurons and those in other layers is their apical dendrite morphology. In most L6 neurons, the apical dendrite does not branch in layer 1, except in claustrum-projecting neurons, and instead typically sends shorter, sparser tuft branches toward layer 4 [4,6]. Although the apical dendrites of L6 pyramidal neurons share several similarities with those in layers 2/3 and 5, their roles in cortical circuits likely differ substantially [6]. These morphological and active property differences indicate that the L6 pyramidal neurons integrate inputs and convey outputs in distinct ways. Therefore, if a cortical area exhibits hemispheric anatomical asymmetry, as in V1 [7], such structural differences may be reflected in the action potential (AP) properties of the same neuronal subtype (e.g., CT pyramidal neurons) when comparing left and right hemispheres within the same region.

APs in pyramidal neurons are initiated at the axon initial segment (AIS), propagating forward to synaptic terminals and backward to the soma and dendrites [8,9,10]. Cortical pyramidal neurons exhibit distinct somatic AP properties, particularly their sharp onset rapidity and variable threshold, attributed to multiple mechanisms [11,12,13], and most prominently the resistive coupling theory [14]. This theory describes how the interaction between AP initiation at the AIS and the somatodendritic load shapes the somatic AP waveform. Specifically, the size difference and short distance between the soma and AIS contribute to the sharp somatic AP observed in cortical neurons. However, AP generation is influenced by several factors, including voltage-gated ion channel types and densities, AIS length and position, dendritic branch geometry, and passive cable properties [10,15,16]. Importantly, these properties are dynamic and can change with repetitive activity. For example, the AP initiation site can shift during repetitive firing, while sustained excitation can enlarge apical dendritic spines and alter ion channel densities. Nevertheless, how these properties collectively influence AP generation and propagation remains elusive.

V1 exhibits hemispheric asymmetry, with documented functional and structural differences between the left and right hemispheres [7,17]. This asymmetry was extensively studied both in normal and pathological conditions, such as in schizophrenia, in which cell density and size were compared between the groups [18,19,20]. However, whether this asymmetry is reflected in the intrinsic electrophysiological properties of a neuronal subtype, such as pyramidal neurons, within the same brain region has not been directly examined. Because AP properties are shaped by morphological and molecular factors that are themselves subject to activity-dependent changes, hemispheric differences in neuronal activity may differentially influence AP waveform properties in pyramidal neurons. For example, the AP initiation site can shift during repetitive firing [21,22], whereas sustained excitation can enlarge apical dendritic spines and alter ion channel densities [23,24]. Thus, baseline differences in AP parameters would be expected between left- and right-hemisphere neurons. At the same time, if apical dendrite truncation alters the somatodendritic contribution to spike generation in a comparable manner, truncated neurons would be expected to exhibit changes in AP parameters in the same direction relative to intact neurons in both hemispheres.

Here, using intracellular recordings from L6 CT pyramidal neurons in the mouse’s primary visual cortex, two primary questions were investigated: (1) whether AP properties of L6 CT pyramidal neurons differ between the left and right hemispheres, and (2) whether neurons with truncated apical dendrites differ from those with intact apical dendrites in AP properties, and whether this difference varies by hemisphere. To address these questions, AP shape properties evoked by 1 s and 3 ms square pulse stimulation were compared between pyramidal neurons with truncated and intact apical dendrites within each hemisphere, and between pyramidal neurons with intact apical dendrites across the two hemispheres.

## 2. Materials and Methods

### 2.1. Electrophysiological Recordings Source

The electrophysiological recordings are obtained from the Allen Brain Atlas [25]. The intracellular recordings are from layer 6a of the primary visual area of mice [26]. At the time of analysis, the initial dataset included recordings from 115 spiny neurons, typically excitatory cells, from several transgenic lines (such as Ntsr1-Cre GN220, Rbp4-Cre KL100, and others), including nine from transgenic-negative cell reporters. For this study, the targeted neuron population was the Ntsr1-labelled neurons. The Ntsr1-Cre GN220 line selectively labels CT pyramidal neurons confined to L6 [27]. The Ntsr1-labelled neurons contributed the largest cohort, comprising 43.3% (n = 46) of the total sample. Accordingly, the recordings analyzed here represent CT pyramidal neurons, constituting the largest group among L6a spiny neurons in the database. Furthermore, neurons that fired only one or two action potentials regardless of stimulus strength were excluded (n = 7), as this firing pattern is often associated with CC pyramidal neurons [4]. Also, one neuron had an average AP width greater than 3 ms, more than three times the overall mean AP width for the dataset. Including this neuron did not affect the conclusions for most parameters and only influenced the AP width comparison. Nevertheless, this neuron was excluded to minimize the risk of including neurons from a different population, given that AP width has high importance for classifying neuron types [28,29]. Therefore, 8 neurons were excluded, and the final dataset used in this study comprised 38 CT pyramidal neurons. These neurons were divided into four groups based on their apical dendrite status (intact Vs truncated), and the brain hemisphere (left vs. right). The final dataset for analysis included 9 intact and 4 truncated neurons from the right hemisphere, and 16 intact and 9 truncated neurons from the left hemisphere.

The recordings in this study were originally obtained in vitro from acute mouse visual cortex slices using whole-cell patch-clamp in current-clamp configuration. In some neurons, the primary apical dendrite extended beyond the boundaries of the acute brain slice and was, therefore, cut during tissue sectioning; these neurons were classified as having truncated apical dendrites and were included in the electrophysiological analysis of spiny neurons in Gouwens et al. (2019) [26]. As stated above, there were 4 truncated neurons from the right brain hemisphere and 9 truncated neurons from the left-brain hemisphere. All neurons, whether with intact or truncated apical dendrites, were stimulated with brief depolarizing current pulses (3 ms) and longer depolarizing current steps (1 s), enabling the characterization of both action potential waveform properties and sustained firing properties under standardized conditions. Detailed experimental procedures are described in the Allen Brain Atlas documentation and the original publication [25,26].

### 2.2. AP Parameters Quantification

The neural responses to short (3 ms) pulse and long (1 s) current steps were obtained from truncated and intact pyramidal neurons in both hemispheres. For each neuron, the first 50 action potentials were used to compute AP parameters, as in our previous studies [29,30]. In the left hemisphere, three neurons, two with intact apical dendrites and one truncated, fired fewer than 50 APs (32, 36, and 48 spikes, respectively). The voltage was interpolated to a resolution of Δt = 1 μs using MATLAB’s (R2022b, MathWorks, Natick, MA, USA) spline function and filtered with MATLAB’s smooth data function (Savitzky-Golay filter, w = 300) to reduce noise. The AP shape was characterized based on four parameters: AP amplitude, AP width, threshold, and rapidity [29]. In short, the AP threshold was defined as the potential at which the membrane potential first-time derivative exceeds 25 mV/ms [11,12]. The AP amplitude was calculated as the difference between the AP peak and threshold, and the width as the full-width of the half-maximum AP amplitude. Finally, the rapidity is calculated as the inverse of the full width at half maximum of the membrane potential second-time derivative [29].

### 2.3. Statistical Analyses

Statistical analyses were performed in MATLAB R2022b. AP parameters were assessed for normality using the Shapiro–Wilk test, and non-parametric methods were selected due to skewed distributions. Group differences were evaluated with Mann–Whitney U tests, and effect sizes were calculated using the rank-biserial correlation (r). Data are reported as mean ± SD, and the results were considered statistically significant at *p* < 0.05. Significance level was adjusted for multiple comparisons using the Bonferroni correction.

## 3. Results

Intracellular recordings from L6a CT pyramidal neurons in the mouse’s primary visual cortex were analyzed. Each neuron was stimulated with both short (3 ms) and long (1 s) current pulses to assess AP waveform properties.

**Figure 1:**
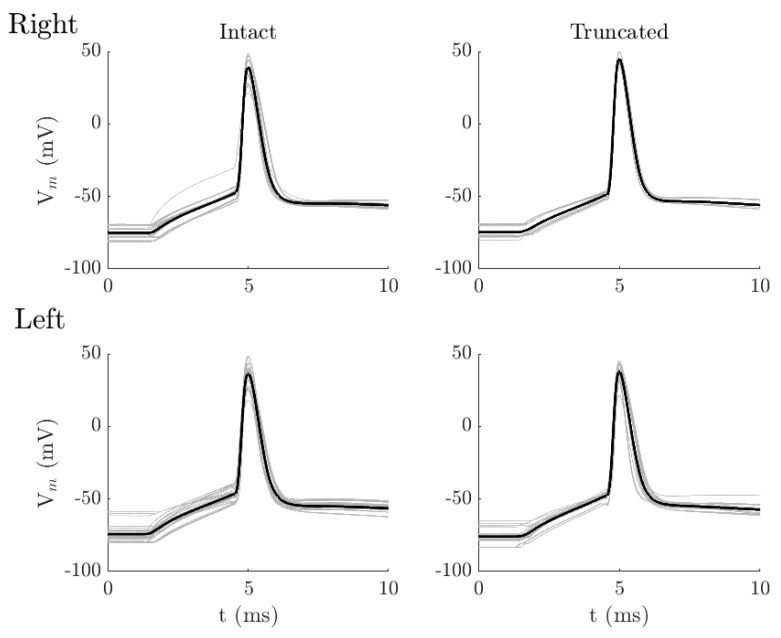
Comparison between APs evoked by a short pulse (3 ms) of the rheobase current pulse. Top figures show the APs from L6a CT pyramidal neurons, with intact (**left**, n = 9) and truncated (**right**, n = 4), in the right-brain hemisphere. Bottom figures show the APs from L6a CT pyramidal neurons, with intact (**left**, n = 16) and truncated (**right**, n = 9), in the left-brain hemisphere. Gray traces indicate APs from all neurons, and black traces represent the average AP.

For a short (3 ms) stimulus, the AP shape was qualitatively similar across all groups, regardless of apical dendrite integrity or hemisphere. Figure 1 shows the AP shapes for all four categories in response to a short 3 ms rheobase current. AP amplitude and width were nearly identical in each hemisphere, with or without an apical dendrite. The AP amplitude was 81.3 ± 9.9 mV for intact neurons and 82.8 ± 8.8 mV for truncated neurons in the left hemisphere (3 ms stimulus; Mann–Whitney U, Z = 0.31, *p* = 0.76, and r = 0.06), and 84.4 ± 14.7 mV for intact neurons and 92.9 ± 2.2 mV for truncated neurons in the right hemisphere (3 ms stimulus; Mann–Whitney U, Z = 0.84, *p* = 0.40, and r = 0.26). The AP width was approximately 0.67 ms for all categories, with no significant differences. Similarly, the AP initiation parameters, rapidity, and threshold also showed no significant differences and only small effect sizes between intact and truncated neurons in both hemispheres. Therefore, the AP shape parameters remained consistent across all categories in response to a short pulse stimulus (Table 1). However, the truncated neurons tended to initiate AP at a higher current level than intact neurons. In the right hemisphere, the average current was 710 ± 196 pA for intact neurons and 817 ± 86.7 pA for truncated neurons (Mann–Whitney U; Z = 1.23, *p* = 0.21, and r = 0.34). A similar pattern was observed in the left hemisphere, with intact neurons requiring 655 ± 176 pA, and truncated neurons needing 744 ± 202 pA (Mann–Whitney U, Z = 0.96, *p* = 0.33, and r = 0.19). Nonetheless, no statistically significant differences were found between the intact and truncated neurons in either hemisphere.

Spikes elicited in response to prolonged rheobase current (1 s current step) remained consistent across groups. In the left hemisphere, the average minimum current required to elicit an AP was 179 ± 99 pA for intact neurons and 161 ± 108 pA for truncated CT pyramidal neurons (Mann−Whitney U; Z = 0.34, *p* = 0.73, and r = 0.07). In the right hemisphere, the corresponding values were 145 ± 68 pA and 175 ± 38 pA (Mann−Whitney U; Z = 0.78, *p* = 0.43, and r = 0.22), respectively. Furthermore, firing frequency was also assessed following around a 50% increase in current amplitude above the rheobase current. Across all conditions, firing frequency averaged approximately 16 Hz (left hemisphere: 15.9 ± 5 Hz for truncated, 16.8 ± 5.7 Hz for intact; right hemisphere: 16.0 ± 4.9 Hz for truncated, 16.7 ± 5.2 Hz for intact), with no significant differences observed between groups. Thus, no significant differences were found in the rheobase current or firing frequency between truncated and intact neurons in response to a 1 s stimulus. However, the shape of the action potentials differed significantly between the two groups.

**Figure 2:**
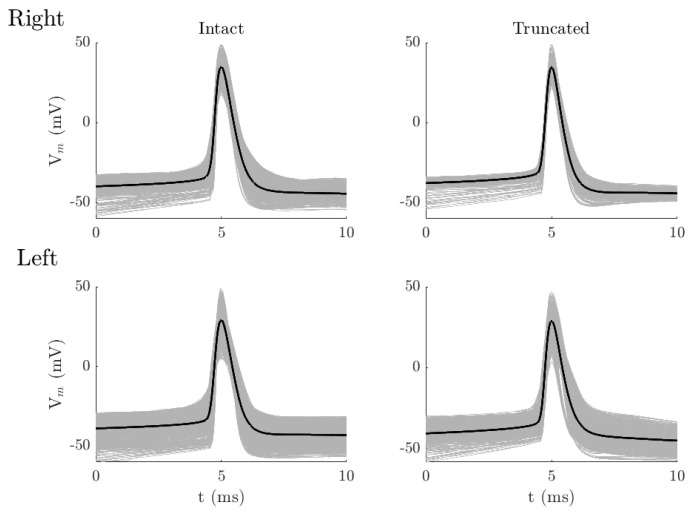
Comparison between APs in spike trains evoked by a 1 s step current pulse. Top figures show the APs from L6a CT pyramidal neurons, with intact (**left**, 9 neurons) and truncated (**right**, 4 neurons), in the right-brain hemisphere. Bottom figures show the APs from L6a CT pyramidal neurons, intact (**left**, 16 neurons) and truncated (**right**, 9 neurons), in the left-brain hemisphere. Gray traces indicate APs from all neurons, and black traces represent the average AP.

Differences in AP shape parameters became more pronounced across the four groups during a 1 s current step. Figure 2 presents a comparison of AP waveforms between intact and truncated neurons in both hemispheres. In the right hemisphere, intact pyramidal neurons fired more rapidly and at lower thresholds compared to truncated pyramidal neurons. For instance, the average AP threshold in the intact neurons was –34.4 ± 3.3 mV, with an average rapidity of 5.7 ± 1.1 ms^−1^, whereas the truncated neurons exhibited a threshold of –31.7 ± 3.0 mV and a rapidity of 5.1 ± 0.9 ms^−1^ (Mann−Whitney U; Z = 11.9, *p* < 0.0001, r = 0.47 for threshold, Z = 7.6, *p* < 0.0001, r = 0.30 for rapidity). In addition, AP amplitude and width were both greater in intact neurons. The comparison between all AP parameters revealed a significant difference using a non-parametric Mann−Whitney test (Table 2). Moreover, the variation of AP parameters was slightly higher in the intact neurons than in the truncated neurons, as measured by the coefficient of variation (standard deviation divided by the mean). The largest variation was observed in AP rapidity, with a coefficient of variation of approximately 19% in the intact neurons versus 17% in the truncated neurons. A similar trend was observed for AP threshold (10% in intact vs. 9% in truncated). However, as shown in Table 1, the variation was comparable in the two groups.

Similar results were observed in the left hemisphere, where all AP parameters showed statistically significant differences between groups. Interestingly, the pattern of differences between neurons with intact and truncated apical dendrites was reversed compared to the right hemisphere. As shown in Table 3, truncated neurons exhibited higher average rapidity than neurons with intact apical dendrites, with an average increase of approximately 16% (Mann−Whitney U test; Z =12.3, *p* < 0.0001, and r = 0.35). The AP threshold, on average, was more hyperpolarized in truncated neurons compared to intact neurons (Mann-Whitney U test; Z = 6.5, *p* < 0.0001, r = 0.19), opposite to what is observed in the right hemisphere. Truncated neurons fire at an average rapidity of 6.5 ± 1.7 $ms^{-1}$ at a more hyperpolarized potential (−34.5 ± 4.9 mV), compared to intact neurons that have an average rapidity of 5.6 ± 1.3 $ms^{-1}$ and a threshold of -32.9 ± 6.2 mV. AP width was similar between the two groups, with slight differences (~1%), although the difference was significant with a very small effect size (Mann-Whitney U test; Z = 3.1, *p* < 0.02, r = 0.08). Furthermore, the variation of AP parameters was also comparable between intact and truncated neurons, in which the largest difference in coefficient of variation was less than 5%.

**Figure 3:**
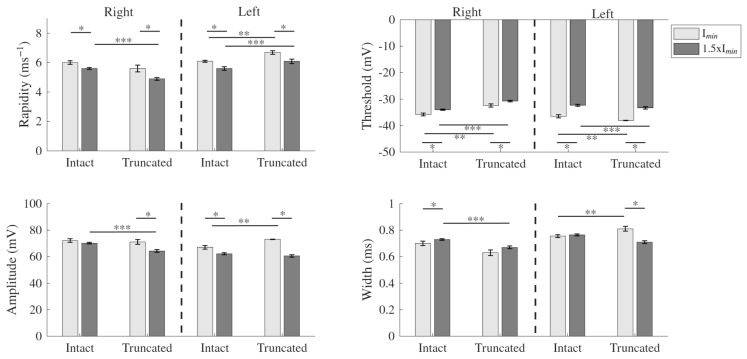
Comparison of AP parameters across the four categories, recorded at the minimum 1 s current to evoke an AP (I_min_) and 50% increase from I_min_ ($1.5I_{\min}$). Bars represent mean ± SE for rapidity, threshold, amplitude, and width. Most comparisons show significant differences (Mann−Whitney U test; * denotes the difference between AP parameters evoked at $I_{\min}$ and 50% above $I_{\min}$ for the same neuron group, ** and *** denote the difference between intact and truncated neurons within the same hemisphere at $I_{\min}$ and 1.5$I_{\min}$, respectively).

The impact of stimulus strength was analyzed. In a previous study, we showed that as stimulus strength increases, AP amplitude and rapidity decrease, while threshold and width increase [31]. To examine whether these patterns persist with and without apical dendrites, the four categories, in response to the rheobase current and around a 50% increase from that value, were compared. In both the intact and truncated neurons, the AP parameters changed in the same direction with increasing stimulus; rapidity and amplitude decreased, and threshold increased (i.e., more depolarization was required to evoke a spike). AP width increased with higher stimulus in the right hemisphere but remained nearly unchanged in the left. Specifically, in the left hemisphere, AP width in the intact neurons stayed consistent at approximately 0.76 ms for both stimulus levels, while in the truncated neurons, it increased from 0.71 ms at rheobase to 0.81 ms with the higher stimulus. While most AP parameters followed similar trends with increasing stimulus strength, except for width in the left hemisphere, the effects of apical dendrite truncation also varied by hemisphere. Figure 3 illustrates these AP parameter changes in response to the increased stimulus in both hemispheres.

Finally, differences in AP shape parameters of the intact CT pyramidal neurons between the two hemispheres were analyzed. The AP amplitude and threshold differed significantly between hemispheres, with the right hemisphere showing lower thresholds and larger amplitudes (Mann−Whitney U test; Z = 9.1, *p* < 0.0001, r = 0.26 for threshold and Z = 14.9, *p* < 0.0001, r = 0.43 for amplitude). In contrast, AP rapidity and width did not differ significantly between hemispheres. AP width was nearly identical (right: 0.73 ± 0.10; left: 0.74 ± 0.10, Mann−Whitney U test; Z =1.32, *p* = 0.19, and r =0.04), as was AP rapidity (right: 5.66 ± 1.06; left: 5.62 ± 1.28, Mann−Whitney U test; Z = 1.62, *p* = 0.11, and r = 0.05).

## 4. Discussion

In this study, the effects of apical dendrite truncation on AP shape properties in CT pyramidal neurons located in layer 6a of the mouse visual cortex, across both the right and left hemispheres, were examined. The results showed that, at a short rheobase current pulse, AP shape parameters were comparable across all four groups: intact and truncated neurons in either hemisphere. Similarly, during a 1 s current stimulus, firing frequency remained consistent among groups, although truncated neurons required a slightly higher current to reach threshold compared to intact neurons; but, the difference was not statistically significant. However, an analysis of the AP shape properties evoked during the 1 s stimulus revealed significant differences between intact and truncated neurons, with dendritic truncation exerting opposing effects on AP shape depending on the hemisphere.

Hemispheric differences were intensively studied in multiple scales, from structural features such as neuron size and density to functional lateralization in response to complex tasks, in regions such as the temporal cortex, motor cortex, and visual cortex [7,32,33,34]. The difference was found to be reflected in the total of neurons between the two sides in some brain areas. For instance, the total number of neurons in the left hemisphere was found to be larger than the right in area 44 of Broca’s area, a region well known for its role in lateralized language processing [35]. However, less is known about the electrophysiological difference between neurons in the two hemispheres. Here, an attempt to examine these differences in AP shape parameters in layer 6a of the CT pyramidal neurons of the primary visual cortex was made. The results showed that AP amplitude and threshold were significantly different between the left and right hemispheres, with a small and medium effect size, in which pyramidal neurons in the right hemisphere produced a larger amplitude and lower threshold compared to the left hemisphere. However, AP width and rapidity were similar in both hemispheres with no significant differences. AP width and rapidity were found to be good indicators in classifying neuronal cell types [28,29]. Thus, while there are some differences, the results indicate that the electrophysiological properties of the intact pyramidal neurons in layer 6a shared some similarities between hemispheres.

Second, the effect of apical dendrite truncation was analyzed in each hemisphere and yielded opposite outcomes. Layer 6 pyramidal neurons typically possess well-developed apical dendritic tufts [4]. In the right hemisphere, neurons with truncated apical dendrites exhibited a mean spike threshold that was 2.7 mV more depolarized, and an approximately 10% slower onset rapidity compared to neurons with intact apical dendrites. This observation is consistent with findings that a larger dendritic surface area increases AP onset rapidity [13]. Similar trends have been reported in human layer 2 and 3 pyramidal neurons, which display faster AP onset and more depolarized thresholds than their mouse counterparts [36]. These interspecies differences have been attributed to the larger dendritic trees in human neurons—about three times the size of those in mice [37]. Thus, truncation of the apical dendrite likely reduces the dendritic load, slowing AP onset and depolarizing the threshold, as observed in the right hemisphere. Interestingly, this resembles effects reported after axonal transection, where AP rapidity decreases and threshold increases, although the underlying mechanisms are likely distinct [38].

In contrast, apical dendrite truncation in the left hemisphere produced opposite effects. These neurons generated action potentials with a 14% faster onset and a threshold approximately 2 mV more hyperpolarized than those with intact dendrites. AP amplitude increased, whereas width remained unchanged. This pattern aligns with findings from studies in L5 cortical pyramidal neurons, where dendritic pinching (equivalent to partial apical dendrite removal) led to more hyperpolarized thresholds, increased amplitude, and nearly constant AP width [39]. However, unlike those results, no significant difference in input resistance was observed here between intact and truncated neurons in either hemisphere. This discrepancy may reflect methodological differences, as the study compared pre- and post-occlusion recordings from the same neuron, whereas the present data derive from different cells.

Changes in dendritic size are known to influence both excitability and AP onset dynamics. AP waveform is strongly determined by the properties of the AIS, such as its location, length, and channel density, and the coupling resistivity between the AIS and the soma-dendritic compartment [14,40,41,42]. Because of this coupling, altering the dendritic load is expected to modify the somatic AP. However, the exact impact of removing part of an apical dendrite on excitability can vary, depending on factors like AIS properties, the types and distributions of ion channels, and potential compensatory mechanisms. For instance, a study found that AIS distance and dendritic morphology jointly establish the resistive coupling between the AIS and the soma. By varying the dendritic diameter and AIS distance, neurons can increase or decrease the somatic AP first-time derivative and shift the AP threshold [43]. This suggests that the covariation of AIS and dendritic properties acts as a homeostatic structural mechanism to stabilize somatic APs across morphological diversity [43]. Thus, even modest morphological differences can produce substantial electrophysiological effects. For example, the length of the apical dendrite drives the differences in excitability between layer 5 pyramidal neurons in the primary and medial secondary visual cortices [44]. Therefore, the observed differences in how apical dendrite truncation affects AP properties in left versus right hemisphere V1 pyramidal neurons may reflect significant underlying differences in morphology or channel distribution between the two hemispheres. However, further studies are needed to fully examine these hemispheric electrophysiological differences.

The opposing effects of apical dendrite truncation on action potential properties across hemispheres may reflect underlying hemispheric asymmetries in neuronal structure and function. Dendrites have been shown to modulate neuronal excitability in a stimulus-dependent manner, acting as passive loads under weak excitation and as active loads under stronger stimulation [39]. However, comparisons of APs evoked at rheobase and at 50% above rheobase yielded similar outcomes, suggesting that stimulus intensity does not account for the hemispheric differences observed here. An alternative explanation may stem from cellular-level asymmetries between hemispheres, as previous studies have reported hemispheric differences at that scale. For instance, layer 3 pyramidal neurons in the rat prefrontal cortex possess longer apical dendrites in the right hemisphere compared to the left [45], and spine density on proximal basal dendrites also varies between hemispheres [46]. Additionally, pyramidal neurons can exhibit intrinsic lateralization that fluctuates with stress and circadian state [46,47]. Hippocampal pyramidal neurons have also been shown to have greater dendritic length in the left hemisphere [48]. Evidence of hemispheric asymmetry in pyramidal cell density in the human dorsolateral prefrontal cortex has also been reported, with a pattern that is altered in individuals with schizophrenia [49]. These studies indicate that the morphological asymmetry of pyramidal neurons is a widespread feature across some brain regions, and emphasize the importance of analyzing neuronal electrophysiological properties separately for each hemisphere, as combining data from the two hemispheres may obscure meaningful differences.

This study has several limitations. First, recordings from L6 spiny neurons were obtained from open-source databases (Allen Brain Atlas), so the sample size for each group was fixed and could not be increased. The left-hemisphere sample was larger than the right-hemisphere sample, and the number of truncated neurons in the right hemisphere was particularly small (n = 4). Although this sample size is comparable to prior studies (typically n = 3–4) [38,39], the small sample size limits the statistical power analysis and increases the risk of Type I and Type II error. A formal prospective power analysis was not feasible, as this study represents a secondary analysis of an existing dataset with a predetermined number of available recordings. Accordingly, the findings from the right-hemisphere truncated group should be preliminary, and replication with larger samples is warranted. Furthermore, although neurons classified as truncated in the dataset were based on a standardized criterion, whether the primary apical dendrite was cut during tissue sectioning, variability in the exact location and extent of the truncation across individual neurons cannot be excluded as a contributing factor to the observed differences, even though there is no indication that the slicing procedure differed systematically between hemispheres. In addition, while the database includes recordings from other transgenic lines targeting L6a spiny neurons, the analyzed neurons were exclusively from the Ntsr1 line to minimize genetic variability. Ntsr1-positive pyramidal cells are largely restricted to L6 in V1 and represent one of the most abundant excitatory populations in L6 V1 [50]. Although CT pyramidal neurons are predominant among Cre-expressing cells in Ntsr1-Cre GN220 mice, a small population of non-CT neurons also expresses Cre [27], and, therefore, the analyzed dataset could include other subtypes of L6 pyramidal neurons. Second, the analyzed L6 pyramidal neurons may include multiple electrophysiological subtypes. Previous electrophysiological classifications in the mouse’s visual cortex have identified several distinct neuronal subtypes [26]. In this study, only neurons with a tonic firing pattern, which is consistent with the CT pyramidal neurons’ spiking pattern, were included, and excluded neurons that fired only one or two action potentials regardless of stimulus strength, a pattern often associated with CC pyramidal neurons [4]. Nonetheless, without direct confirmation of neuron class for each recorded cell, some heterogeneity may remain.

## 5. Conclusions

Overall, this study shows that apical dendrite integrity exerts hemisphere-specific effects on somatic AP properties in L6a CT pyramidal neurons, despite a broadly similar baseline excitability between left and right hemispheres. Intact neurons displayed comparable AP rapidity and width but differed in threshold and amplitude, whereas dendritic truncation produced significant and opposing changes in all AP parameters within each hemisphere. These findings suggest that the mechanisms linking dendritic morphology to AP generation might differ between hemispheres, implying that CT neurons in the left and right V1 may implement distinct rules for integrating dendritic load. While acknowledging the limits to generalizing these results, the findings provide new insight into neuronal activity, including potential hemispheric differences and the influence of apical dendrites on AP generation. Further studies will be needed to elucidate the mechanisms underlying these effects.

## Figures and Tables

**Table 1:** AP parameters of L6a CT pyramidal neurons from both hemispheres in response to a short 3 ms current stimulus.

	Right Hemisphere		Left Hemisphere	
	Intact (n = 9)	Truncated (n = 4)	Intact (n = 16)	Truncated (n = 9)
${\mathrm{IFWd}}^{2}\;(ms^{-1})$	7.4 ± 1.5	6.3 ± 0.7	6.8 ± 1.1	7.2 ± 1.2
Threshold (mV)	−46.2 ± 6.3	−48.2 ± 1.7	−46.3 ± 3.7	−47.4 ± 2.8
Amplitude (mV)	84.4 ± 13.9	92.9 ± 2.1	81.3 ± 9.6	82.8 ± 8.3
Width (ms)	0.67 ± 0.07	0.67 ± 0.04	0.67 ± 0.06	0.66 ± 0.09

**Table 2:** AP parameters of L6a CT pyramidal neurons from the right hemisphere in response to a 1 s current pulse.

Right	Intact (n = 9)	Truncated (n = 4)
${\mathrm{IFWd}}^{2}\;(ms^{-1})$	5.7 ± 1.1	5.1 ± 0.9 *
Threshold (mV)	−34.4 ± 3.3	−31.7 ± 3.0 *
Amplitude (mV)	71.5 ± 8.4	68.0 ± 8.1 *
Width (ms)	0.73 ± 0.10	0.66 ± 0.08 *

Mean ± SD, * *p* < 0.05, *p*-value is from Mann−Whitney U test.

**Table 3:** AP parameters of L6a CT pyramidal neurons from the left hemisphere in response to a 1 s current pulse.

Left	Intact (n = 16)	Truncated (n = 9)
${\mathrm{IFWd}}^{2}\;(ms^{-1})$	5.6 ± 1.3	6.5 ± 1.7 *
Threshold (mV)	−32.9 ± 6.2	−34.5 ± 4.9 *
Amplitude (mV)	63.6 ± 11.1	65.1 ± 10.5 *
Width (ms)	0.74 ± 0.10	0.73 ± 0.13 *

Mean ± SD, * *p* < 0.05, *p*-value is from Mann-Whitney U test.

## Data Availability

The electrophysiological data used in this study were obtained from the Allen Cell Types Database, and can be found here: https://celltypes.brain-map.org/data (accessed 19 January 2026).

## Abbreviations

The following abbreviations are used in this manuscript:

CT	corticothalamic pyramidal neurons
CC	corticocortical pyramidal neurons
V1	Primary Visual Cortex
L6	Layer 6
IFWd^2^	inverse of the full width at half maximum of the membrane potential second-time derivative
